# Soil Quality Assessment of Different Land Use Types Based on TOPSIS Method in Hilly Sandy Area of Loess Plateau, Northern China

**DOI:** 10.3390/ijerph192417059

**Published:** 2022-12-19

**Authors:** Yida An, Lei Zhang, Qing Wang, Yunwei Han

**Affiliations:** 1 School of Environment and Resource, Southwest University of Science and Technology, Fucheng District, Mianyang 621010, China; 2 School of Economics and Management, Taiyuan Normal University, Yuci District, Jinzhong 030619, China; 3 Department of Tourism, Taiyuan University, Xiaodian District, Taiyuan 030032, China

**Keywords:** soil quality, land use types, ecological risk, kastanozems, weighted TOPSIS method, Loess Plateau

## Abstract

In order to combat land desertification and to evaluate the soil quality of different cover land types, and thereby determine the best land use strategy for vegetation restoration, this research comprehensively analyzed the soil quality of different land use/cover types in the hilly sandy area of the Loess Plateau by using the Kruskal–Wallis test (K–W test) and principal component analysis (PCA) technique for order preference by the similarity to ideal solution (TOPSIS), and the potential ecological risk index (*RI*). The result indicated that the cropland abandonment over a period of time could improve the soil quality to a certain extent; however, the process of natural restoration was very slow compared with that of the planted mixed shrubs. The soil quality of all land use/cover types in the hilly sandy area of Youyu County clearly improved after 10–25 years of revegetation, but the degree of improvement varied greatly with the different planted species and their combinations. The low levels of soil organic matter, total nitrogen and available phosphorus were the major limiting factors affecting soil quality improvement under different land use/cover types, not metal toxicity. Pioneer species of *H. rhamnoide*, *C. korshinskii* and *Pinus sylvestris* var. *mongolica*, etc. are vital to vegetation restoration of the study area. Revegetation using these species is therefore recommended to combat future desertification in this area.

## 1. Introduction

Soil, as a natural and nonrenewable resource, has attracted worldwide attention with the development of populations, and has become the most fragile ecosystem due to long-term cultivation by humans. As the problems exposed in the coordinated development among environments, resources and populations become more obvious, the issue of soil quality is attracting worldwide attention, as soil resources are a kind of non-renewable and fragile resource in the human time scale [1,2]. Land use by humans is a vital and direct activity that affects soil quality. Human beings intervene and adjust the direction of the soil biogeochemical cycle and the redistribution process of surface substances through different utilization methods, thus changing soil quality. Reasonable land use can improve soil structure and enhance the resistance of soil to external environmental changes [3,4]. However, unreasonable land use will lead to the degradation of soil quality, increased soil erosion, and a decrease in biodiversity [5,6].

As the carrier of various ecological processes in ecosystem, the nutritional status of soil is one of the key indexes to measure the restoration and maintenance of ecological functions in degraded ecosystems [7]. Different types of vegetation greatly affect the formation and development of soil. Understanding the response of soil nutrients to land use change is of great significance for understanding the soil fertility of each community and the cycle mechanism of nutrient elements [8,9]. Many studies have also focused on vegetation restoration, soil nutrients and enzyme activities, soil fertility and heavy metal fixation [10,11,12,13,14]. In addition, with the increasing prevalence of farmers going out of business, the impact of long-term abandoned cropland on the environment has also attracted worldwide attention [15,16]. At present, the effective development of vegetation restoration and ecological construction has become an urgent scientific issue, especially for regions with poor ecological environment.

Soil quality is defined as “the ability of soil to play a role in natural or managed ecosystems to maintain the productivity of animals and plants, while ensuring the healthy life of human beings” [17,18]. Soil quality can reflect the level of soil management, and it is also of great significance to the restoration of degraded land, regional land resource management and sustainable land use, which has become area of increasing concern [19,20,21]. At present, there are many methods for evaluating soil quality [22,23,24,25,26] and there is no unified evaluation standard of soil quality due to the different evaluation objectives and scales of evaluation objects and the complexity of evaluation work [27]. The principal component analysis (PCA) method, as a classic feature extraction method, simplifies the original high-dimensional variables and preserves the original data information to the maximum extent [28,29,30]. Andrews et al. [31] and Yemefack et al. [32] proposed the minimum data set (MDS) method based on the highest factor loading value in PCA, which has been widely recognized. The technique for order preference by similarity to ideal solution (TOPSIS) method has no special requirements on sample size and is not interfered with by the selection of reference sequences, so it is widely used in water quality evaluation and other fields, but seldom used in soil quality [33,34].

The research area of Youyu County in Shanxi Province, China is located at the southern fringe of Mu US Desert and Hobq Desert, which is the transitional zone of forest to desert, humid to arid, plain to plateau, coastal to inland, monsoon to non-monsoon and agricultural to animal husbandry. Youyu County suffered seriously complex erosion by wind and water before the 1950s, and since the early 1950s, the local government has carried out a long-term revegetation strategy for combating desertification. After more than 70 years of revegetation, the vegetation coverage across the entire county increased from 0.26% to 56% today, which has achieved the transformation from ecological deficit to ecological surplus [35], and now it is become a typical national-level demonstration area in China for combating desertification in the hilly sandy area of the Loess Plateau, where soil erosion and desertification are interlaced [36]. The serious soil erosion before 1950 in this area not only led to river siltation, but also caused severe desertification, while unreasonable land use was the main reason for the aggravation of water and soil loss and the deterioration of the ecological environment [37,38]. An in-depth analysis of the response of soil chemical properties to different land use/cover types and soil quality evaluation can provide a scientific basis for ecological restoration and sustainable development in hilly sandy areas of the Loess Plateau. The study hypothesized that the soil quality in the study area would be varied under different revegetation types. Based on the above reasons and hypothesis, this study aimed to: (1) evaluate the soil quality under different cover types, and (2) clarify the ecological risk of soil heavy metals in order to determine the best land use strategy.

## 2. Overview of the Study Area

Youyu County (39°41′–40°17′ N, 112°07′–112°38′ E), located in northern Shanxi Province, at the northeast margin of Loess Plateau, was selected as the study area. The area covers a total area of about 1967 km^2^, and belongs to the loess hilly region, with temperate semi-arid continental monsoon climate and four distinct seasons. The annual average temperature is about 3.7 °C, the annual average precipitation is about 360 mm, and the frost-free period is 100–120 d. The soil in this area is mainly chestnut soil and aeolian sandy soil, which has a coarse texture and poor anti-erosion ability, and is vulnerable to wind erosion, water erosion and desertification [39].

**Figure 1:**
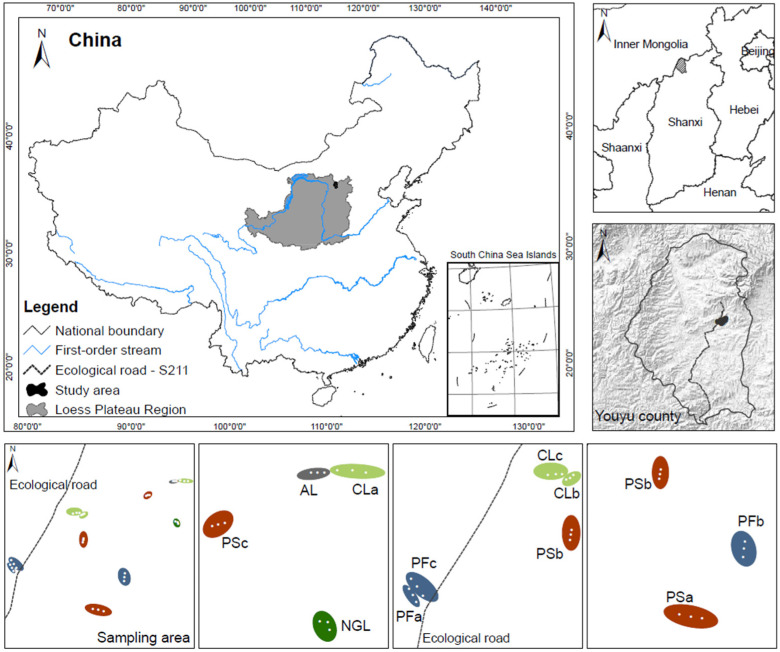
Location of the study area at Youyu County of Shanxi Province in the Loess Plateau and sampling sites. Note: CL (Croplands); AL (Abandoned land); NGL (Natural grassland); PSa, PSb, PSc (Planted shrublands—*Caragana korshinskii*, *Hippophae rhamnoides*, *Caragana korshinskii* and *Hippophae rhamnoides* mixed forest); PFa, PFb, PFc (Planted woodlands—*Populus simonii*, *Pinus sylvestris* var. *mongolica*, *Populus simonii* and *Pinus sylvestris* var. *mongolica* mixed forest).

The zonal vegetation in this area belongs to a temperate steppe–forest transition zone [40] or grassland. Natural vegetation is mainly grassland composed of *Stipa capillata*, *Thymus mongolicus* and *Artemisia mongolica*; shrubs such as *Hippophae rhamnoides* and *Caragana korshinskii* are distributed on both sides of the river; the planted forest is mainly composed of *Populus simonii* and *Pinus sylvestris* var. *mongolica*. According to the actual situation of local land use, the following five major different types of land use including 11 cover types were determined (Table 1). Croplands (CL), planted woodlands (PW), planted shrublands (PS), abandoned land (AL) and natural grassland (NGL) were selected as sample sites (Figure 1).

## 3. Methodology

### 3.1. Soil Sampling

The collection of soil samples was collected from Youyu County in September 2018. A total of 11 sample sites with different cover types were selected from five land use types across the Youyu county, and three samples with a size of 10 m × 10 m were randomly selected from each sample site, for a total of 33 samples (Table 1). The sizes of the quadrats were determined by considering the minimum community areas of each vegetation type [41] and the species–area curve [42]. 0.5 kg of 0–10 cm mixed soil sample were packed and repeated three times in each site to measure the soil chemical properties.

### 3.2. Laboratory Analysis

The methods for measuring the soil chemical properties have been described by Liu [43]. Soil organic matter (OM) was measured by the potassium dichromate volumetric method under an external heat source (NY/T1121.6-2006). Soil total nitrogen (TN) was measured using the Kjeldahl method (NY/T53-1987). Soil available nitrogen (AN) was measured by the 1.0 mol/L NaOH-H3BO3 alkaline hydrolysis diffusion method (LY/T1 229-1999). Soil available potassium (AK) was extracted by 1.0 mol/L NH4OAc and measured by atomic absorption spectrophotometry (NY/T889-2004). Soil available phosphorus (AP) was extracted by 0.5 mol/L NaHCO3 and measured by ultraviolet visible spectrophotometry (NY/T1121.7-2006). Soil available metals (ACu, AMn, AFe and AZn) were measured by atomic absorption spectrophotometry after being extracted by DTPA (NY/T890-2004). Measurement of soil Hg and As were conducted by atomic fluorescence spectrometry (NY/T1 121.10-2006; NY/T1 121.11-2006). Soil Cd and Pb were extracted by 1.0mol/L HNO3 and measured by graphite furnace atomic absorption spectrometry (GB/T17141-1997). Measurement of soil Cr was obtained by flame atomic absorption spectrophotometry (HJ491-2009). Soil pH was measured by potentiometric analysis (NY/T1 121.2-2006). In order to ensure the reliability of the results, triplicate measurements were conducted; and the averages and standard deviations were calculated.

### 3.3. Data Processing

The data were pretreated using Excel 2007 (Table A1 and Table A2). Figures were made using ArcGIS and Origin software. The Kruskal–Wallis test (K–W test) was conducted using SPSS 16.0 software to determine the differences in soil chemical properties under different cover types. PCA and correlation analyses between soil parameters were used to establish MDS, and the TOPSIS method was used to evaluate soil quality under different cover types, which were achieved by using R. RI [44] was used to evaluate the current status of soil heavy metal pollution in the study area.

#### 3.3.1. K–W test

The K–W test (a non-parametric test) was used to test the differences of soil indexes among different sample groups [45]. This method was used as it is equivalent to ANOVA, but does not apply the normal distribution hypothesis of ANOVA, and the sample size for each group in the study was relatively small (n = 3). The main step of the K–W test was to sequence all data of specific soil parameters in each group and calculate the H-statistic (for details see Equation (1)). Multiple comparisons using the Bonferroni method [46,47] were made in order to determine the differences within each group under a soil parameter (*p* = 0.05):

(1) $$H=\frac{12}{n_{t}\left(n_{t}+1\right)}\overset{m}{\underset{i=1}{\sum }}\frac{R_{i}^{2}}{n_{i}}-3\left(n_{t}+1\right)$$

where, *H* is the test statistic, $n_{t}$ is the total observed value of an index for all sample groups, *n_i_* (*i* = 1, 2, …, m) is the number of observations for group *i*, $R_{i}^{2}$ is the square of rank sum for group *i*.

#### 3.3.2. Establishment of MDS

First, the K–W test was used to test the differences of soil parameters and the PCA method was used to analyze the parameters which with differences (*p* = 0.05). By calculating the loading of each soil parameter on all principal components (PC) with eigenvalues ≥ 1, which with loading ≥ 0.5 on the same PC were divided into one group. Once a soil parameter had a high loading on more than one PC, it was merged into the group with low correlation with the others, the norm value of soil parameters in each group was calculated, and parameters within the range of 10% of the highest norm values were selected for the minimum data set (MDS). When there was only one high loading soil parameter in a PC, it was then directly entered into the MDS. Once there was more than one high loading parameter retained in a PC, Pearson’s correlation analysis was used to determine whether these parameters could be considered redundant. If the correlation coefficient between the parameters was relatively high (r ≥ 0.5), the soil parameter with the highest norm value was determined to enter MDS, otherwise each soil parameter enters into MDS [48,49]. The norm value was calculated by Equation (2).

(2) $$N_{ik}=\sqrt{\overset{k}{\underset{1}{\sum }}\left(u_{ik}^{2}\lambda_{k}\right)}\;$$

where, $N_{ik}$ is the norm value of soil parameter *i* on PCs which eigenvalues ≥ 1, $u_{ik}$ is the loading of soil parameter *i* on PC *k*, and $\lambda_{k}$ is the eigenvalue of PC *k*.

#### 3.3.3. Soil Quality Evaluation Based on TOPSIS

TOPSIS is a practical method for ranking and selecting a number of possible alternatives by measuring Euclidean distances. It evaluates the samples according to the relative distances between positive and negative ideal solutions [50]. The steps of TOPSIS method are as follows.

Step 1. Normalization of the Soil Parameter

Since the soil parameters have different dimensions and orders of magnitude, which need to be standardized, where which are divided into positive and negative parameters. The normalization method is as follows.

(3) $$\mathrm{Positive}\;\mathrm{soil}\;\mathrm{parameters}:\;\;y_{ij}=\frac{x_{ij}-min\left(x_{ij}\right)}{\max\left(x_{ij}\right)-min\left(x_{ij}\right)}$$

(4) $$\mathrm{Negative}\;\mathrm{soil}\;\mathrm{parameters}:\;y_{ij}=\frac{\max\left(x_{ij}\right)-x_{ij}}{\max\left(x_{ij}\right)-min\left(x_{ij}\right)}$$

where, $y_{ij}$ is the standardized value of the parameter; $x_{ij}$ is the initial value of the parameter; $\max\left(x_{ij}\right)$ and $min\left(x_{ij}\right)$ are the maximum and minimum values of each soil parameter, respectively.

Step 2. Weight Determination of Soil Parameter

Entropy weight method is a decision-making method that quantifies and synthesizes multi-factor information objectively, assigns values to each factor and determines the influence degree of each factor on the objective [51,52]. The application of EW method can effectively take into account the degree of variation of the soil parameters and objectively reflect its importance. It was calculated by Equation (5).

(5) $$e_{i}=-k\overset{n}{\underset{j=1}{\sum }}f_{ij}\ln f_{ij}$$

(6) $$w_{i}=\frac{1-e_{i}}{m-\sum_{i=1}^{m}e_{i}}\;$$

where, $e_{i}$ is the information entropy of the soil parameter *i* among the *m* soil parameters and *n* evaluation objects; i=1,2,…,m; $f_{ij}=y_{ij}/\overset{n}{\underset{j=1}{\sum }}y_{ij}$, k=1/lnn; $w_{i}$ is the weight of soil parameter *i*, $0\leq w_{i}\leq 1$, $\overset{m}{\underset{i=1}{\sum }}w_{i}=1$.

Step 3. Determination of Positive and Negative Ideal Solutions

The positive and negative ideal solutions were detailed by Equations (7) and (8).

(7) $$\mathrm{Positive}\;\mathrm{ideal}\;\mathrm{solutions}:\;Z^{+}=\left\{max(y_{ij})|i=1,2,\ldots ,m\right\}=\left\{\alpha_{1}^{+},\alpha_{2}^{+},\ldots ,\alpha_{m}^{+}\right\}\;\;$$

(8) $$\mathrm{Negative}\;\mathrm{ideal}\;\mathrm{solutions}:\;Z^{-}=\left\{min(y_{ij})|i=1,2,\ldots ,m\right\}=\left\{\alpha_{1}^{-},\alpha_{2}^{-},\ldots ,\alpha_{m}^{-}\right\}$$

Step 4. Calculate the Distance to the Positive and Negative Ideal Solutions

The Euclidean distances from each alternative to the positive and negative ideal solutions were calculated by Equations (9) and (10).

(9) $$D_{j}^{+}=\sqrt{\overset{m}{\underset{i=1}{\sum }}w_{i}{\left(\alpha_{ij}^{+}-\alpha_{ij}\right)}^{2}}\;$$

(10) $$D_{j}^{-}=\sqrt{\overset{m}{\underset{i=1}{\sum }}w_{i}{\left(\alpha_{ij}^{-}-\alpha_{ij}\right)}^{2}}\;$$

where, $D_{j}^{+}$ and $D_{j}^{-}$ is the Euclidean distance of evaluation object *j* to the positive and negative ideal solutions, respectively; $\;\alpha_{ij}^{+}$ and $\alpha_{ij}^{-}$ are the standardized values in the positiveand negative ideal solutions, respectively; $\;w_{i}$ is the weight of parameter *i*, which was calculated by step 2.

Step 5. Calculation of Soil Quality Index (SQI)

(11) $$SQI_{j}=\frac{D_{j}^{-}}{D_{j}^{+}+D_{j}^{-}}\times 100\;$$

where, $SQI_{j}$ is the soil quality index of soil sample j, the range of $SQI_{j}$ is [0, 100], a larger SQI value indicated better soil quality.

#### 3.3.4. Potential Ecological Risk Index

The method of potential ecological risk index proposed by Hakanson [44] was applied to evaluate five soil heavy metals (Cr, Cd, Pb, Hg and As) pollution. The method considered the content of heavy metals in soil, and factors such as multi-element synergy, toxicity level, pollution concentration and the sensitivity of the environment to heavy metal pollution [53,54,55], which has practical significance to evaluate the status of soil heavy metal pollution in the study area. It was calculated by Equation (12).

(12) $$RI=\overset{n}{\underset{i=1}{\sum }}E_{i}=\overset{n}{\underset{i=1}{\sum }}T_{i}\cdot Cr_{i}/Cs_{i}$$

where, $E_{i}\;$ is the potential ecological risk index of heavy metal *i*; $T_{i}\;$ is the corresponding toxicity coefficient; $Cr_{i}\;$ is the measured concentration value (mg/kg^−1^) of heavy metal *i*, $Cs_{i}$ is the background value of soil heavy metal *i* in the region [56]; RI is the potential ecological risk index of multiple heavy metals. The division criteria of potential ecological risk and heavy metal toxicity coefficient see Table A3.

## 4. Results

### 4.1. Soil Chemical Properties under Different Land Use/Cover Types

**Figure 2:**
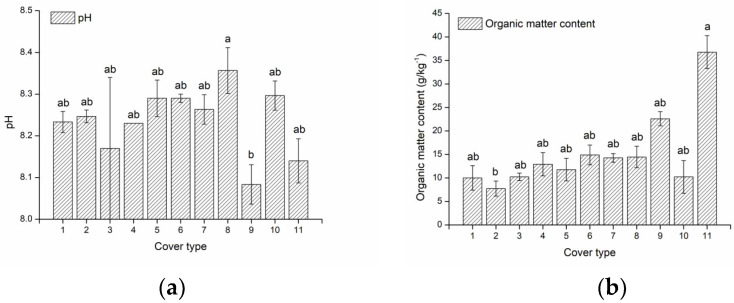

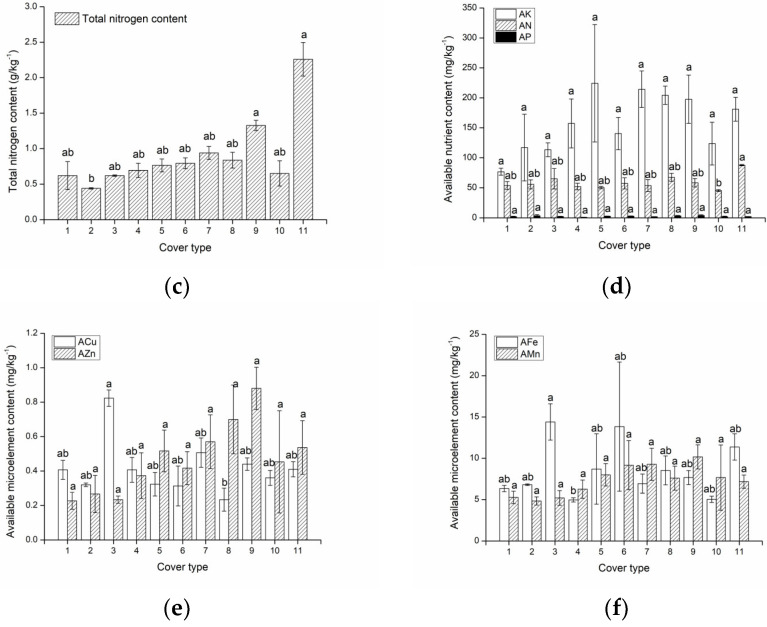
Soil chemical characteristics under different cover types. (**a**) pH level; (**b**) Organic matter content (g/kg^−1^); (**c**) Total nitrogen content (g/kg^−1^); (**d**) Available nutrient content (mg/kg^−1^); (**e**,**f**) Available microelement content (mg/kg^−1^). Statistical significance is denoted by differing letters, different letters meant there was significant difference among groups (*p* < 0.05).

According to the classification standard of soil pH, the pH values of all sample groups in the study area were alkaline (7.5–8.5). Based on the Nutrient grading standards for the second National Soil Survey [57], the soil organic matter (OM) content of natural grassland (NGL) was the highest at grade 2 (30–40 g/kg^−1^), followed by that of *Hippophae rhamnoides* (H.R)-*Caragana korshinskii* (C.K) mixed forest was grade 3 (20–30 g/kg^−1^). The soil OM content of the cropland where *Zea mays* (Z.M) planted was the lowest at grade 5 (6–10 g/kg^−1^), and that of other cover types was grade 4 (10–20 g/kg^−1^). The mixed forest had higher soil OM content than pure forest. The pattern of soil OM and TN content was basically the same under different land use types, which ordered from high to low was: NGL > planted shrublands (PS) > planted woodlands (PW) > abandoned land (AL) > croplands (CL) (Figure 2a–c).

In terms of soil available nutrients, the content of total nitrogen (TN) in NGL was significantly higher than that in AL (*p* = 0.01), while the difference between other cover types was not significant (50.33–67.33 mg/kg^−1^). However, the ANOVA indicated that there was no significant difference of soil available phosphorus (AP) and potassium (AK) contents under different cover types (Figure 2d).

According to the grading standard (5-level of very lack, lack, moderate, rich and very rich) for soil available microelement content in China summarized by the existing research [58], the soil ACu content of all cover types were moderate. Soil AZn content was moderate in all cover types of PS, NGL, very lack in all cover types of cropland and lack in the rest types. The soil AFe content in *Fagopyrum esculentum* (F.E), *Populus simonii* (P.SC) + *Pinus sylvestris* var. *mongolica* (P.SM) mixed forest and NGL was rich, while that in other cover types was moderate. Except for the lack AMn content in the cropland where *Zea mays* was planted, it was moderate under other cover types (Figure 2e,f). It could be found that the available microelement of all PS were in the moderate grade, which has best overall grade with NGL (moderate—rich), which was superior to PW.

By the horizontal comparison of each soil parameter between cropland planted with different crops and AL [59], the responses of each soil parameter to the cropland abandonment were obtained (Table 2). After the cropland was abandoned, the change amplitude of soil ACu content was the largest (68.6%), while the change of pH was the smallest (1%). In addition, the effects of cropland abandonment on soil chemical properties were mainly reflected in the contents of available nutrients, including AZn, AMn, AFe, AK and AN. The content of soil nutrients increased after cropland abandonment was (from high to low): AZn > AMn > AK > TN > OM, and that decreased was (from high to low): AFe > AN > ACu > AP.

### 4.2. Evaluation of SQI under Different Land Use/Cover Types

Based on the K–W test (Equation (1)), all soil parameters were significantly different under different cover types except for Hg and As (H (OM) = 24.62, P (OM) = 0.01; H (TN) = 25.97, P (TN) = 0.00; H (AN) = 20.32, P (AN) = 0.03; H (AP) = 20.15, P (AP) = 0.03; H (AK) = 22.60, P (AK) = 0.01; H (ACu) = 23.87, P (ACu) = 0.01; H (AZn) = 21.66, P (AZn) = 0.02; H (AFe) = 23.07, P (AFe) = 0.01; H (AMn) = 20.33, P (AMn) = 0.03; H (Cr) = 27.30, P (Cr) = 0.00; H (Cd) = 22.29, P (Cd) = 0.01; H (Pb) = 23.21, P (Pb) = 0.01). Soil quality under 11 cover types was further appraised using PCA and TOPSIS methods and based on the measured soil parameters (Table 3); the scores of four principal components (PC-1, PC-2, PC-3, PC-4) were extracted by the PCA with an eigenvalue ≥ 1.0 and their cumulative contribution rate reached 85.97% (Table 3). This indicated that the four principal component scores accounted for most of the variability in soil quality under the 11 land cover types.

**Figure 3:**
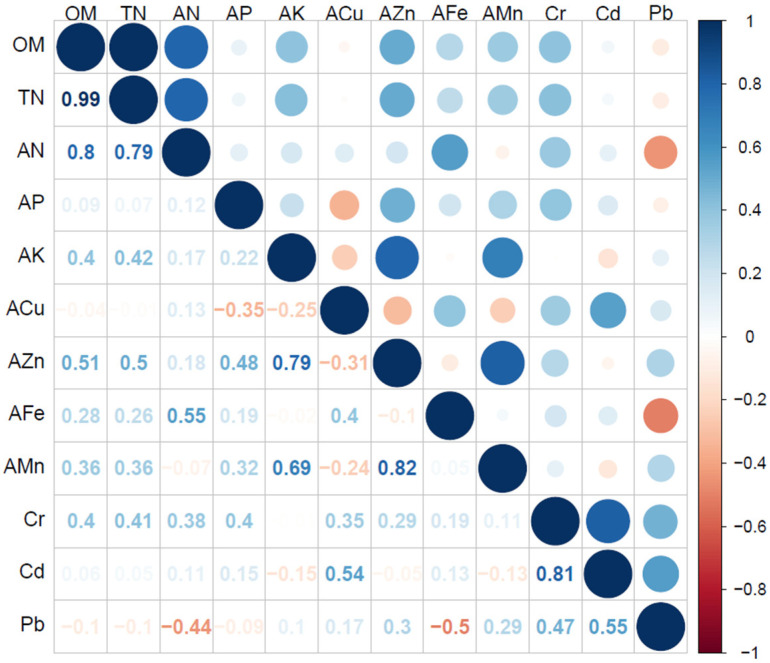
Correlation coefficients matrix between each soil parameters. Note: OM (organic matter), TN (total nitrogen); the shadow circle in the right upper region represents the direction and degree of interaction between soil parameters, the blue and red shadow represents positive and negative correlation, respectively; the left lower half is the correlation coefficient between parameters; the deeper the shadow soil parameters the greater positive/negative correlation between the two functions.

Among edaphic parameters under the 11 cover types, soil OM, TN, AN, AN and AZn had the higher factor loading in PC-1, and norm values all met the criteria of 10% of the highest (1.846), however, due the significant correlation between TN, AN and OM (*p* < 0.01), OM and AZn entered MDS. Among the soil parameters met, the factor loading criteria on PC-2, Cr had the highest norm value (1.652), and there was a significant correlation between Cd and Cr (*p* < 0.01), so Cr and AMn entered MDS. In PC-3, Cd had the highest norm value (1.530), and there was no significant correlation with Pb, thereby Cd and Pb entered MDS. AP directly entered MDS since only which met the factor loading criteria in PC-4, the correlation matrix between soil parameters is shown in Figure 3. Overall, there were seven MDS parameters for soil quality evaluation: OM, AP, AZn, AMn, Cr, Cd and Pb, the weight of each parameter entering MDS was calculated according to Equations (3)–(6) (Table 3), which indicated that soil OM, TN and AP were the major factors affecting soil quality. In total, these results were consistent with the mentioned soil property results.

Based on the TOPSIS method, the soil quality index (SQI) and its ranking under 11 cover types in the study area were calculated according to Equations (7)–(11) (Table 4). The SQI ranged from 18.6 to 59.8, and ordered from high to low was: S.C+T.M+A.M > H.R > H.R+C.K > P.SM > P.SC + P.SM > C.K > A.N > P.SC > AL > Z.M > F.E., that is, *Stipa capillata* + *Thymus mongolicus* + *Artemisia mongolica* (S.C + T.M + A.M) > *Hippophae rhamnoides* (H.R) > *H. rhamnoides* + *Caragana korshinskii* (H.R + C.K) > *Pinus sylvestris* var. *mongolica* (P.SM) > *Pinus sylvestris* var. *mongolica* + *Populus simonii* (P.SC + P.SM) > *C. korshinskii* (C.K) >*Avena nuda* (A.N) > *P. simonii* (P.SC) > abandoned land (AL) > Z.M (*Zea mays*) > *Fagopyrum esculentum* (F.E). That is, the order of average SQI under different land use/cover types from high to low was: natural grassland (NGL) > planted shrublands (PS) > planted woodlands (PW) > abandoned land (AL) > croplands. This result indicated that the soil quality of different land use/cover types were different in the hilly sandy area of Youyu County, China. The natural grassland, planted shrubs of *H. rhamnoide* or mixed *H. rhamnoide* and *C. korshinskii*, and the planted forest of *Pinus sylvestris* var. *mongolica* in the study area had the best soil improvement effects, while the cropland for *Fagopyrum esculentum* and *Zea mays* planting, abandoned land and the planted *P. simonii* pure forest had the lowest effects on soil quality improvement.

### 4.3. Ecological Risk Evaluation of Soil Heavy Metal Pollution

Table 5 showed the comparison of soil heavy metal contents in the study area with those of other standards. Soil Cr, Pb, Cd, and Hg contents were lower than the background average values in the area where they located, and As contents were higher than the background values. All metals contents were much lower than the risk screening values of soil pollution in China agricultural lands (GB 15618-2018).

According to Equation (12), Table 5 and Table A3, the RI and the potential ecological risk index $E_{i}$ of five heavy metal elements in the study area were calculated (Table A4). According to the heavy metal pollution classification standard [44], the results indicated that $E_{i}$ values of Cr, Cd, Pb and As in all cover types were lower than 40, which pertains to a low potential ecological risk. The $E_{i}$ of Hg was at a moderate potential pollution risk (higher than 40 and less than 80) in all the other cover types, except for less than 40 in the mixed forest of *Populus simonii* and *Pinus sylvestris* var. *mongolica* (P.SC + P.SM). The average values of RI (H (RI) = 18.831, P (RI) = 0.04) of heavy metals under different cover types were all at the standard of low ecological risk (RI < 150. For soil heavy metal pollution risk, Hg had a moderate potential ecological risk under all cover types except *P. sylvestris* var. *mongolica* + *P. simonii* (P.SC + P.SM) mixed forest. Soil Cr, Cd, Pb and As were at low potential ecological risk under all cover types, and the overall potential ecological risk index of multiple heavy metals (RI) was at low ecological risk, which indicated that metal toxicity was not the major limiting factor affecting soil quality improvement under different land use/cover types in the study area.

## 5. Discussions

### 5.1. The Response of Soil Properties to Conversion of Cropland to Abandoned Land

Soil OM as a vital source of nutrients, it promotes the formation of soil aggregates [62]. The soil TN and OM content increased in the abandoned cropland. Due to the lack of accumulation of stubble on the surface of cropland, coupled with the effects of cultivation, soil OM decomposed rapidly, the released nitrogen was absorbed and assimilated by crops in large quantities while serious losses in due to leaching occurred, which resulted in low TN content in the surface soil [37,63,64]. The result showed that soil AK content increased, while AP and AN content decreased. Except for causes from human disturbances, this might be due to the high content of K in the loess parent material of the soil in the study area, and the consumption of soil AP and AN caused by the gradual growth of natural vegetation. Although the mobility soil of AK is relatively high [65], soil leaching is relatively weak due to the limitations of local climatic conditions (precipitation). The content, distribution and availability of soil microelements are not only related to soil parent materials, but also affected by human factors such as farming systems and fertilizer applications. According to the existing research, the increase of AZn and AMn content and the decrease of AFe and ACu content in soil after the cropland was abandoned, AFe had significant seasonal dynamics, indicating that the long-term single or combined application of phosphorus and potassium fertilizers in the crop growth process improved the content of AFe and ACu in soil, but had no significant effect on the content of AZn [66,67].

On the other hand, the ecological risk evaluation showed that there was no significant difference in the Ei of all heavy metals before and after cropland abandonment, while the soil quality index (SQI) of cropland was relatively poor. Overall, cropland abandonment has a certain effect on soil quality. Vegetation systems in natural succession after cropland abandonment have a strong function in nutrient enrichment, which is vital in maintaining the sustainable use of land. In addition, this study showed that cropland abandonment after 10–12 years could improve the soil quality to a certain extent, but the process of natural restoration of abandoned cropland was very slow compared with the planting of mixed shrubs of *Hippophae rhamnoides* and *Caragana korshinskii* (H.R + C.K) 10 years after initial planting. As a typical semiarid area of Youyu County located on the southern fringe of Mu US Desert and Hobq Desert, the slow natural restoration of cropland abandonment may easily increase the risk of natural hazards such as soil erosion, floods and desertification [68]. Therefore, in the initial ecological restoration of abandoned cropland, a revegetation process with human assistance is the best choice for soil improvement in the study area, which can be achieved many decades sooner than natural revegetation; a similar result has been reported by Mohan [69].

### 5.2. Soil Quality under Different Land Use/Cover Types

As the cover with the best soil improvement effect, natural grassland (NGL) had the highest soil quality of all the cover types. The high content of soil OM and TN in NGL might be due to the dense roots of natural herbaceous plants, which provides a large amount of organic matter and nutrients for the topsoil (0–10 cm). In addition, sheep manure produced by herding sheep in this area can improve the content of soil OM and TN to improve soil properties [70]. Some of them are replenished to the soil by atmospheric bulk deposition [71]. However, some studies in adjacent areas have concluded that grassland soil nutrients were the worst [72], which it might be due to overgrazing. Although it was suggested in some studies that the grassland ecosystem has a certain elasticity to grazing [73], the damage to soil and vegetation is still difficult to recover [74] and seriously affects the physicochemical properties of surface soil [75], especially for the arid and semi-arid regions with relatively fragile ecological environments [76]. Therefore, in the conversion of cropland to natural grassland and the development of animal husbandry, the grazing intensity should be regulated and mixed agriculture should be encouraged, which is comprised of animal husbandry and traditional agriculture balanced development.

The contents of available P and K in the planted forests were the highest, which was due to the abundance of surface litter and less artificial disturbance. As a fast-growing forest, *Populus simonii* was the vegetation with the worst soil quality in planted shrubs and forests (PS and PF). The soil quality of *H. rhamnoides* (H.R.) and *Pinus sylvestris* var. *mongolica* (P.SM) pure forest were the highest in PS and PF, respectively. The soil quality of mixed forests was in the middle level. According to the RI ranking above, the advantages of H.R. and P.SM pure forest were mainly reflected in the content of heavy metals, and the soil nutrient content of mixed forest was relatively high. This is due to the surface litter and soil animal diversity being more abundant than those in the pure forest [77], and the litter breaking up and infiltrating into the soil along with the precipitation, thus accelerating the decomposition and release of nutrient elements [78,79]. In summary, natural grasslands, planted shrubs and mixed forests should be emphasized during the ecological construction in this region in the future, and large area plantings of *Populus simonii* (P.SC) pure forest should be avoided due to their heavy consumption of more soil nutrients and water during later growth. Therefore, the mixed *P. simonii* and *Pinus sylvestris* var. *mongolica* forest is critical in improving the degraded *P. simonii* pure forest. A similar result has been reported by Liang et al. [80].

As a land use type with poor soil quality, cropland seriously consumed soil nutrients. The ANOVA also showed that the soil OM and TN contents in cropland cropped with *Zea mays* were significantly lower than that in the natural grassland growing *Thymus mongolicus, Stipa capillata* and *Artemisia mongolica* (S.C+T.M+A.M). This was due to the cropland crops absorbing large amounts of soil nutrients in the growth process, with the crop tillage and harvest accelerating the decomposition of soil OM [81]. Usually, the soil in northern China is deficient in nitrogen, and these crops were basically nitrogen-loving crops in the study area; successive planting has resulted in serious nitrogen consumption in the soil. In addition, agricultural production has a significant effect on soil microelement consumption, with a small compensation. Therefore, the implementation of returning cropland to grassland in this area can lead to greater ecological environment benefits. In order to improve the soil fertility of the existing farmland and increase the crop yield [82], local materials should be advocated where the straw of the crushed corn and other crops should be directly returned to the field. In addition, due to the low ecological risk of heavy metals in the study area, the development of ecological agriculture should be emphasized on the basis of ensuring food security [83].

## 6. Conclusions

The study indicated that the cropland abandonment over a period of time could improve the soil quality to a certain extent; however, the process of natural restoration was very slow compared with that of the planted mixed shrubs, indicating that the revegetation process with human assistance is the best choice for improvement of the soil quality in the study area.

After 10–25 years of revegetation, the soil quality of all land use/cover types in hilly sandy area of Youyu County clearly improved, but the degree of improvement varied greatly with the different planted species and their combinations. The natural grassland of *Stipa capillata* + *Thymus mongolicus* + *Artemisia mongolica*, planted shrublands of *H. rhamnoide* or mixed *H. rhamnoide* and *C. korshinskii* and the planted woodlands of *Pinus sylvestris* var. *mongolica* in the study area had the best soil improvement effects, while the croplands for *Fagopyrum esculentum* and *Zea mays* plantings, and the abandoned land and planted *P. simonii* pure forestlands had the lowest effects on soil quality improvement.

The low levels of soil organic matter, total nitrogen and available phosphorus were the major limiting factors affecting soil quality improvement under different land use/cover types, not metal toxicity.

Pioneer species of *H. rhamnoide*, *C. korshinskii* and *Pinus sylvestris* var. *mongolica*, etc. play important roles on vegetation restoration in the study area. Revegetation using these species is therefore recommended to combat future desertification in this area.

## Figures and Tables

**Table 1:** The basic information of the 11 sample sites in the study area.

No.	Land Use Type	Cover Type	Recovery Years(a)	Altitude (m)
1	Croplands (CL)	A.N	-	1470–1472
2		Z.M	-	1387–1390
3		F.E	-	1378–1386
4	Planted woodlands (PW)	P.SC	25	1426–1428
5		P.SM	15	1375–1380
6		P.SC + P.SM	10	1376–1383
7	Planted shrublands (PS)	C.K	10	1425–1431
8		H.R	15	1390–1392
9		H.R + C.K	10	1461–1469
10	Abandoned land (AL)	S.C (rare)	10–12	1472–1474
11	Natural grassland (AGL)	S.C + T.M + A.M	-	1518–1520

Note: A.N (*Avena nuda*); Z.M (*Zea mays*); F.E (*Fagopyrum esculentum*); P.SC (*Populus simonii*); P.SM (*Pinus sylvestris var. mongolica*); C.K (*Caragana korshinskii*); H.R (*Hippophae rhamnoides*); S.C (*Stipa capillata*); T.M (*Thymus mongolicus*); A.M (*Artemisia mongolica*).

**Table 2:** Response of soil properties to conversion of cropland to abandoned land.

Soil Parameter	Content	Coefficient of Variation	Range of Variation/%	Mean/%
pH	8.24 ± 0.05	0.01	[0.6, 1.6]	1.0
Organic matter	9.56 ± 1.22	0.13	[−0.1, 32.2]	11.4
Total nitrogen	0.58 ± 0.10	0.16	[4.8, 47.7]	19.1
Available N	54.92 ± 8.08	0.15	[−30.3, −15.5]	−21.5
Available P	2.37 ± 0.76	0.32	[−44.9, −3.5]	−17.8
Available K	107.67 ± 21.10	0.20	[5.7, 61.3]	25.4
Available Cu	0.48 ± 0.23	0.48	[−56.1, 12.5]	−18.6
Available Zn	0.30 ± 0.11	0.36	[66.7, 95.7]	86.0
Available Fe	8.14 ± 4.24	0.52	[−65.1, −20.5]	−37.2
Available Mn	5.74 ± 1.30	0.23	[45.5, 58.8]	50.6

**Table 3:** Principal component load matrix, calculated norm value and weight of soil paramete.

Soil Parameter	Principal Component Load Matrix				Group	Norm Value	Weight (%)	Parameter Direction
	*PC-1*	*PC-2*	*PC-3*	*PC-4*				
OM	**0.875**	0.180	0.248	0.284	1	1.846	21.67	Pos.
TN	**0.871**	0.179	0.240	0.315		1.841		Pos.
AN	0.662	0.490	0.490	0.040		1.725		Pos.
AP	0.420	0.152	0.149	**0.855**	4	1.315	8.82	Pos.
AK	0.660	0.504	0.050	0.116		1.567		Pos.
ACu	0.092	0.750	0.212	0.216		1.308		Pos.
AZn	**0.798**	0.484	0.260	0.039	1	1.831	18.04	Pos.
AFe	0.299	0.528	0.426	0.369		1.308		Pos.
AMn	0.634	**0.530**	0.239	0.012	2	1.583	14.28	Pos.
Cr	0.529	**0.559**	0.554	0.179	2	1.652	11.13	Neg.
Cd	0.151	0.659	**0.670**	0.111	3	1.530	15.47	Neg.
Pb	0.052	0.062	**0.939**	0.288	3	1.488	10.60	Neg.
Eigenvalues	4.017	2.690	2.371	1.239				
% of variance	33.47	22.41	19.76	10.32				
Cumulative variance (%)	33.47	55.89	75.65	85.97				

Note: OM (organic matter), TN (total nitrogen); values in bold font represents parameters retained in MDS.

**Table 4:** Soil quality evaluation result based on TOPSIS.

No.	Land Use Type	Cover Type	*D* ^+^	*D* ^−^	SQI	Ranking
1	Croplands (CL)	A.N	0.305	0.221	42.1	7
2		Z.M	0.359	0.103	22.3	10
3		F.E	0.354	0.081	18.6	11
4	Planted woodlands (PW)	P.SC	0.289	0.144	33.3	8
5		P.SM	0.224	0.234	51.1	4
6		P.SC+P.SM	0.219	0.224	50.6	5
7	Planted shrublands (PS)	C.K	0.230	0.193	45.6	6
8		H.R	0.192	0.252	56.8	2
9		H.R+C.K	0.227	0.271	54.4	3
10	Abandoned land (AL)	S.C	0.292	0.130	30.8	9
11	Natural grassland (NGL)	S.C+T.M+A.M	0.182	0.271	59.8	1

Note: Z.M (*Zea mays*); F.E (*Fagopyrum esculentum*); A.N (*Avena nuda*); C.K (*Caragana korshinskii*); H.R (*Hippophae rhamnoides*); P.SC (*Populus simonii*); P.SM (*Pinus sylvestris var. mongolica*); S.C (*Stipa capillata*); T.M (*Thymus mongolicus*); A.M (*Artemisia mongolica*; *D^+^*, *D^−^* (Euclidean distance of evaluation object to the positive and negative ideal solutions, respectively); SQI (Soil quality index).

**Table 5:** Soil heavy metal concentration of agricultural land (mg/kg^−1^).

Region	Cr	Cd	Pb	Hg	As	References
Youyu County	42.81	0.12	8.62	0.03	10.8	This Study
Background value ^a^	61.8	0.128	15.8	0.027	9.8	[56]
GB 15618-2018 ^b^	250	0.6	170	3.4	25	[60]
Beijing, Tianjin	52.3	0.145	18.7	0.092	7.9	[61]

Note: ^a^ Shanxi soil background value of 0–20 cm; ^b^ Risk screening values for soil contamination of agricultural land (pH > 7.5).

## Data Availability

Not applicable.

## Appendix A

**Table A1:** Soil chemical properties under different cover types.

Land Cover Type	OM (g/kg^−1^)	TN (g/kg^−1^)	AN (mg/kg^−1^)	AP (mg/kg^−1^)	AK (mg/kg^−1^)	pH	ACu (mg/kg^−1^)	AZn (mg/kg^−1^)	AFe (mg/kg^−1^)	AMn (mg/kg^−1^)
1	10.02 ± 2.62	0.62 ± 0.20	53.67 ± 6.66	2.00 ± 0.61	76.67 ± 5.77	8.23 ± 0.03	0.41 ± 0.06	0.23 ± 0.05	6.33 ± 0.38	5.27 ± 0.75
2	7.74 ± 1.61	0.44 ± 0.01	55.67 ± 7.51	3.50 ± 1.90	117.00 ± 55.65	8.25 ± 0.02	0.32 ± 0.01	0.27 ± 0.11	6.80 ± 0.10	4.83 ± 0.49
3	10.24 ± 0.80	0.62 ± 0.01	65.00 ± 16.82	2.03 ± 0.75	113.33 ± 11.55	8.17 ± 0.17	0.82 ± 0.05	0.23 ± 0.02	14.40 ± 2.19	5.20 ± 0.89
4	12.93 ± 2.47	0.69 ± 0.10	52.00 ± 5.57	0.93 ± 0.15	157.33 ± 40.87	8.23 ± 0.00	0.41 ± 0.07	0.37 ± 0.13	4.97 ± 0.25	6.27 ± 1.10
5	11.78 ± 2.40	0.76 ± 0.09	50.33 ± 2.08	2.73 ± 0.42	224.33 ± 98.10	8.29 ± 0.04	0.32 ± 0.07	0.52 ± 0.12	8.70 ± 4.26	8.00 ± 1.35
6	14.90 ± 2.10	0.79 ± 0.08	57.33 ± 9.29	2.93 ± 0.32	140.33 ± 27.02	8.29 ± 0.01	0.31 ± 0.12	0.42 ± 0.10	13.83 ± 7.81	9.17 ± 2.97
7	14.27 ± 0.97	0.94 ± 0.09	53.67 ± 9.87	1.83 ± 0.21	214.33 ± 30.55	8.26 ± 0.04	0.51 ± 0.09	0.57 ± 0.16	6.93 ± 1.16	9.27 ± 1.94
8	14.47 ± 2.29	0.84 ± 0.11	67.33 ± 6.66	3.20 ± 0.80	204.33 ± 15.28	8.36 ± 0.06	0.23 ± 0.07	0.70 ± 0.20	8.53 ± 1.75	7.60 ± 1.45
9	22.60 ± 1.51	1.33 ± 0.07	58.33 ± 7.09	3.93 ± 1.25	197.67 ± 40.41	8.08 ± 0.05	0.44 ± 0.04	0.88 ± 0.12	7.67 ± 0.85	10.17 ± 1.46
10	10.23 ± 3.48	0.65 ± 0.18	45.33 ± 1.53	1.93 ± 1.02	123.67 ± 35.64	8.30 ± 0.04	0.36 ± 0.04	0.45 ± 0.30	5.03 ± 0.38	7.67 ± 3.95
11	36.77 ± 3.50	2.26 ± 0.24	87.67 ± 1.15	2.17 ± 0.06	181.00 ± 20.00	8.14 ± 0.05	0.41 ± 0.04	0.54 ± 0.16	11.37 ± 1.59	7.17 ± 0.80
Mean ± Std.	15.09 ± 8.15 a	0.90 ± 0.50 a	58.76 ± 12.90 b	2.47 ± 1.10 b	159.09 ± 58.71 b	8.24 ± 0.92 b	0.41 ± 0.16 a	0.47 ± 0.23 b	8.60 ± 3.97 b	7.32 ± 2.31 b

Note: OM (organic matter); TN (total nitrogen); the letter “a” and “b” represent the significant difference level of the K–W test (a: *p* ≤ 0.01. b: *p* ≤ 0.05).

**Table A2:** Soil heavy metal content under different cover types.

Land Cover Type	Cr (mg/kg^−1^)	Cd (mg/kg^−1^)	Pb (mg/kg^−1^)	Hg (mg/kg^−1^)	As (mg/kg^−1^)
1	30.67 ± 1.72	0.07 ± 0.00	3.47 ± 1.27	0.03 ± 0.00	10.21 ± 1.23
2	50.57 ± 3.42	0.16 ± 0.04	8.67 ± 1.50	0.03 ± 0.00	10.55 ± 0.73
3	51.13 ± 7.85	0.16 ± 0.06	8.20 ± 6.35	0.03 ± 0.00	11.26 ± 0.80
4	32.67 ± 2.30	0.12 ± 0.05	12.20 ± 2.95	0.04 ± 0.01	11.05 ± 0.44
5	31.67 ± 12.02	0.08 ± 0.00	6.03 ± 2.80	0.03 ± 0.00	11.00 ± 0.32
6	36.37 ± 2.14	0.10 ± 0.03	4.60 ± 2.10	0.03 ± 0.00	11.01 ± 0.17
7	41.07 ± 3.37	0.12 ± 0.03	10.80 ± 1.25	0.03 ± 0.01	11.17 ± 0.55
8	37.80 ± 3.22	0.08 ± 0.01	5.27 ± 3.31	0.03 ± 0.00	11.25 ± 0.17
9	58.07 ± 4.45	0.15 ± 0.00	14.70 ± 1.93	0.03 ± 0.01	10.36 ± 1.30
10	48.63 ± 4.79	0.12 ± 0.01	15.83 ± 1.37	0.03 ± 0.00	9.94 ± 0.61
11	52.23 ± 9.48	0.12 ± 0.06	5.10 ± 2.21	0.03 ± 0.00	10.99 ± 0.34

**Table A3:** Classification standard of *E_i_* and RI and heavy metal toxicity coefficient by Hakanson (1980) [44].

E_i_	Pollution Degree		RI		Risk Degree
E_i_ < 40	Low potential ecological risk		RI < 150		Low ecological risk
40 ≤ E_i_ < 80	Moderate potential ecological risk		150 ≤ RI < 300		Moderate ecological risk
80 ≤ E_i_ < 160	Considerable potential ecological risk		300 ≤ RI < 600		High ecological risk
160 ≤ E_i_ < 320	High potential ecological risk		RI ≥ 600		Very high ecological risk
E_i_ ≥ 320	Very high potential ecological risk				
Item	Cr	Cd	Pb	Hg	As
E_i_	2	30	5	40	10

Note: *E_i_* is the potential ecological risk index of heavy metal *i*; RI is the potential ecological risk index of multiple heavy metals.

**Table A4:** Potential ecological risk of heavy metals under different cover types.

Item	*E_i_*					RI
	Cr	Cd	Pb	Hg	As	
1	0.99 ± 0.06	15.78 ± 0.36	1.10 ± 0.40	42.96 ± 6.46	10.42 ± 1.25	71.26 ± 5.94
2	1.64 ± 0.11	38.36 ± 9.66	2.74 ± 0.47	44.44 ± 3.92	10.77 ± 0.74	97.95 ± 7.97
3	1.65 ± 0.25	37.81 ± 15.08	2.59 ± 2.01	45.43 ± 3.73	11.49 ± 0.82	98.98 ± 10.00
4	1.06 ± 0.07	28.05 ± 10.67	3.86 ± 0.93	65.19 ± 20.04	11.28 ± 0.45	109.43 ± 23.57
5	1.02 ± 0.39	19.69 ± 0.47	1.91 ± 0.89	40.99 ± 2.26	11.23 ± 0.32	74.84 ± 2.86
6	1.18 ± 0.07	23.91 ± 6.70	1.46 ± 0.66	39.51 ± 2.26	11.23 ± 0.17	77.28 ± 5.54
7	1.33 ± 0.11	28.36 ± 7.52	3.42 ± 0.40	49.88 ± 18.88	11.39 ± 0.56	94.38 ± 20.58
8	1.22 ± 0.10	18.28 ± 2.84	1.67 ± 1.05	46.42 ± 4.53	11.48 ± 0.18	79.07 ± 5.38
9	1.88 ± 0.14	36.17 ± 1.08	4.65 ± 0.61	40.00 ± 9.71	10.57 ± 1.33	93.27 ± 10.05
10	1.76 ± 0.17	36.10 ± 4.05	5.39 ± 0.46	59.33 ± 7.57	10.92 ± 0.67	113.50 ± 4.49
11	1.89 ± 0.34	37.00 ± 16.57	1.73 ± 0.75	54.00 ± 2.00	12.07 ± 0.38	106.70 ± 17.32

Note: *E_i_* is the potential ecological risk index of heavy metal *i*; RI is the potential ecological risk index of multiple heavy metals.
